# The Potential for Engineering Enhanced Functional-Feed Soybeans for Sustainable Aquaculture Feed

**DOI:** 10.3389/fpls.2016.00440

**Published:** 2016-04-05

**Authors:** Eliot M. Herman, Monica A. Schmidt

**Affiliations:** School of Plant Sciences, University of ArizonaTucson, AZ, USA

**Keywords:** aquaculture, aquafeed, soybean, carotenoid, vaccines, Kunitz trypsin inhibitor, soybean agglutinin, P34 allergen

## Abstract

Aquaculture is the most rapidly growing segment of global animal production that now surpasses wild-capture fisheries production and is continuing to grow 10% annually. Sustainable aquaculture needs to diminish, and progressively eliminate, its dependence on fishmeal-sourced feed from over-harvested fisheries. Sustainable aquafeed sources will need to be primarily of plant-origin. Soybean is currently the primary global vegetable-origin protein source for aquaculture. Direct exchange of soybean meal for fishmeal in aquafeed has resulted in reduced growth rates due in part to soybean’s anti-nutritional proteins. To produce soybeans for use in aquaculture feeds a new conventional line has been bred termed *Triple Null* by stacking null alleles for the feed-relevant proteins Kunitz Trypsin Inhibitor, lectin, and P34 allergen. *Triple Null* is now being further enhanced as a platform to build additional transgene traits for vaccines, altered protein composition, and to produce high levels of β-carotene an intrinsic orange-colored aquafeed marker to distinguish the seeds from commodity beans and as the metabolic feedstock precursor of highly valued astaxanthin.

## Aquaculture is the Fastest Growing Segment of Global Animal Production that Will Require New Sustainable Sources of Feed

The global human population is projected to grow to 9.6 billion individuals by 2050 and will continue to 12 billion individuals or more by 2100 (Gerland et al., 2014). This population growth will require an increase of 70% more animal production that translates to a need for 235% more animal feed. Soybean is the largest plant-source input of protein and reduced nitrogen for formulated animal feed that drives a global industry of production and consumption. Aquaculture is most rapidly growing sector of the animal food-production sectors, increasing at nearly 10% per year (Food and Agriculture Organization [FAO], 2012). Aquaculture-sourced fish surpasses wild capture fisheries in annual production and this differential is projected to rapidly increase due to overfishing and habitat destruction in parallel to increased production in the aquaculture sector. As aquaculture displaces wild capture fisheries providing feed sources for cultured animals presents an immense challenge. Many commercially raised fish are carnivorous (e.g., trout and salmon), while others are omnivores and herbivores (e.g., catfish, carp, and tilapia); therefore, different fish species vary in their capacity to effectively use different kinds of feed. Aquaculture currently over-relies on fishmeal to provide high-quality feed protein. Annual fishmeal production has been constant for the last 15 years at approximately 6.5 million metric tons. Current or projected increase of fishmeal production to support aquaculture growth cannot be sustained (Watson et al., 2015).

A sustainable feed solution for aquaculture would be for farmed fish to be fed renewable plant-sourced protein and oil products harvested from terrestrial farms (Gatlin et al., 2007; Naylor et al., 2009; Rust et al., 2011). While substituting plant protein for fishmeal is now routine at low levels, in most cases increasing the proportion of plant protein in fish feed limits fish growth rates and feed efficiency. There is an opportunity to engineer seed crops as functional feed ingredient that specifically address the challenges of increasing the proportion of plant-based protein in commercial fish aquafeeds. Soybean is currently used as a primary source of vegetable protein supplement for aquafeed (Tacon et al., 2012) and is a large US export (Hardy, 1996). Soybean, particularly at high inclusion levels, in aquaculture can result in reduced growth rate dues at least in part from soybean’s anti-nutritional proteins (Krogdahl et al., 1994, 2010; Baeverfjord and Krogdahl, 1996; Burrells et al., 1999; Bakke-McKellep et al., 2000; Buttle et al., 2001; Lilleeng et al., 2007; Sahlmann et al., 2013). One approach is to use biotechnology to suppress anti-nutritional proteins or to alter seed protein composition (see Herman, 2003, for review). To some degree conventional breeding and stacking of traits derived from non-biotech sources such as collections and mutation to enhance seed composition can also meet trait goals. Other enhancements that are not encoded within the soybean genome will require adding foreign genes to produce stacked trait soybeans optimized for high-performance feed-ingredient (Herman, 2009).

## Breeding Low Anti-Nutritional Bioactivity Soybeans and its Development as a Platform to Express Additional Transgene Traits

### Developing a Conventional Low Bioactivity Soybean Seed; the Creation of Triple Null

Soybeans null for bioactive seed proteins that have been isolated from the USDA soybean collection include Kunitz trypsin inhibitor (KTI) null (Orf and Hymowitz, 1979; Hymowitz, 1986), soybean agglutinin (SBA) null (Orf et al., 1978; Goldberg et al., 1983) and immunodominant soybean allergen P34 protein nulls (Josephs et al., 2006). Each of these nulls has the potential to partially address concerns of soybean feed/food consumption and stacked together these traits can form a platform for engineering enhanced soybean varieties. A *Triple Null* stack of the recessive nulls of KTI, SBA, and P34 in the standard cultivar *Williams* (the archetype used to elucidate the soybean genome) has been produced (Schmidt et al., 2015a). The parental components of the *Triple Null (p34,kti,le*) stack include frame shift mutants of P34 (Josephs et al., 2006; Bilyeu et al., 2009) and KTI (Jofuku et al., 1989) that still accumulate a small amount of authentic protein due to a shift of the start methionine inward in the open reading frame Schmidt et al. (2015a). The *Lectin* (LE) null results from a large transposon and does not produce a functional transcript or lectin protein accumulation (Orf et al., 1978; Goldberg et al., 1983). Proteomic analysis of *Triple Null* shows that the line lacks this trio of bioactive proteins while retaining the full complement of other proteome constituents without any other collateral bioactive protein alterations (Schmidt et al., 2015a).

### *Triple Null* Soybeans Can Be Transformed to Stack Additional Traits

**FIGURE 1:**
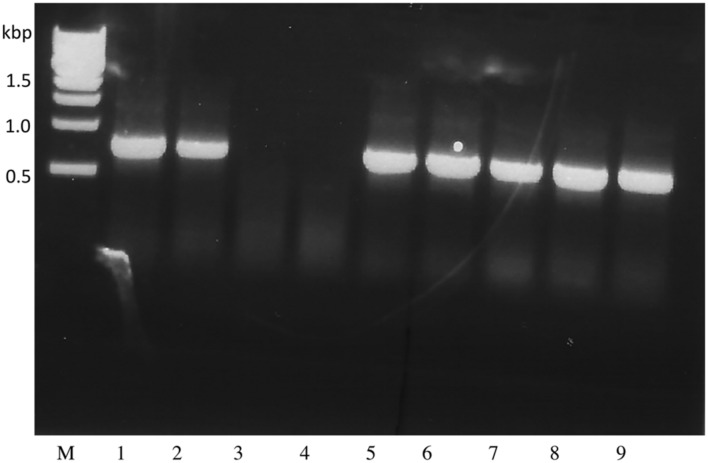
***Triple Null* soybean is transformable via biolistic transformation of somatic embryos.** Genomic PCR was performed on *T*_1_ segregating *Triple Null* plants transformed with the hygromycin resistance marker gene expression cassette. Presence of the PCR amplicon ~ 700 bp in all samples except 3 and 4 indicate the presence of the hygromycin gene in those plants DNA, indicating a stable transformation event that is segregating in an expected Mendelian ratio.

To enable further enhancement of *Triple Null* its capacity for biolistic transformation was assessed. Somatic embryos were induced from *Triple Null* and used in test biolistic transformation studies using a hygromycin-selection cassette (see Schmidt and Herman, 2008; Schmidt et al., 2011, 2015b for methods). The resulting transformed lines were selected and regenerated into somatic embryos that were then germinated to produce a *T*_0_ population of plants. The resulting *T*_1_ seeds are a segregating population typically requiring two or three generations of recurrent selection to produce homozygote lines. **Figure 1** shows PCR analysis of a segregating population assessed by the presence of the *Hygromycin* marker demonstrating that *Triple Null* that is bred into a *Williams 82* background is capable of being transformed by the same protocols as cv *Jack*. This indicates that the traits of *Triple Null* can be used as a platform to stack additional transgene traits and can be exchanged for other standard transformation soybean lines.

## Potential Biotechnology Traits to Enhance *Triple Null’s* Performance as an Ingredient in Aquafeed

The capacity to transform *Triple Null* is enabling for further biotechnological modifications using gene expression and silencing to further optimize performance of the seeds in aquafeeds. As an aquafeed source there are potential targets of expressing bioactive proteins such as vaccines and to further remodel the seed proteome such as by optimizing its protein content for digestibility. There are additional modifications that could be added to soybean that produces products that are not feasible to produce from soybean’s genome. These traits could reproduce lipid-soluble carotenoids and omega fatty acids that would otherwise be obtained from fish oil with the same sustainability issues as fishmeal or from chemical synthesis and/or modification.

### Protein Enhancement

The soybean seed proteome consists of two dominant seed storage proteins (SPs) 7S conglycinin and 11S glycinin and a number of additional moderately abundant proteins that are often bioactive including Kunitz and Bowman Birk trypsin inhibitors, lectin, P34 allergen, urease and lipoxygenases. *Triple Null* stacking alleles from the USDA soybean collection addresses some of the most problematic bioactive proteins for aquafeed (Krogdahl et al., 1994; Buttle et al., 2001; Lilleeng et al., 2007). The SPs comprise about 65% of the seed’s protein content and is regulated by genotype as a breeding trait. Most of the major soybean proteins are members of small gene families (for example Nielsen et al., 1989) making it difficult to identify conventional alleles that that silence all of the individual genes. Biotechnology approaches using co-suppression and RNAi has been used to knockout soybean genes and gene families of β-conglycinin (Kinney et al., 2001; Kim et al., 2014), P34 allergen (Herman et al., 2003), and both β-conglycinin and glycinin (Schmidt et al., 2011). Other lines have reduced Bowman Birk trypsin inhibitor (BBI) content by co-expressing an inactive variant (Livingstone et al., 2007) as not to further reduce the sulfur amino acid content of soybeans.

Silencing major soybean SPs results in compensatory remodeling of the proteome but not the seed protein content. This proteome compensation is termed protein rebalancing (Herman, 2014) that in soybean results when a major SP is silenced by either mutation or targeted genetic modification that should result in a significant shortfall of accumulated seed protein. The soybean remodels its proteome resulting in other intrinsic seed proteins increasing in accumulation to compensate for loss of major proteins that maintains both protein content (Schmidt et al., 2011) and total amino acid composition (Schmidt et al., 2011; Kim et al., 2014). From the biotechnologist’s perspective proteome rebalancing can be exploited as a technology to replace major soybean SPs with other transgene products (Herman and Schmidt, 2014). Suppressing β-conglycinin that comprises 20% of the total seed protein results in enhanced accumulation of glycinin that maintains the standard protein content. Glycinin consists of a small five gene family to test the potential of exploiting the proteome rebalancing process a model GFP gene was constructed as a glycinin mimic and introgressed into β-conglycinin silenced seeds as an additional glycinin allele. This resulted in increasing GFP accumulation eight-fold from 1 to 8% of the total protein (Schmidt and Herman, 2008). Although this appears to be a potential engineering strategy to increase foreign protein content in seeds the proteome rebalancing processes by maintaining protein content and relative total amino acid this strategy does not appear to be effective to increase the sulfur amino acid content of soybean by expressing zein in a β-conglycinin suppressed background (Kim et al., 2014).

### Feed based Vaccines Are Economic Solution to Disease

In animal production animal density is a key economic factor. High animal density is an economic necessity of production and this aggravates the problems of animal competition, waste management, and controlling the potential for disease. For fish production a number of bacterial and viral diseases have emerged that impact production. For many fish species especially the omnivorous and herbivorous species the low individual value of each fish limits the potential expenditure for antibiotic treatment or preventive vaccination. Fish can be immunized orally (Companjen et al., 2005; Plant and LaPatra, 2011; Tobar et al., 2011, 2015) or by bath exposure and if the vaccine antigens are inexpensive and effective this can enable disease prevention. There are problems to be resolved to make these protocols as effective as individual injection immunization particularly with primary sensitization (Munang’andu et al., 2015; Mutoloki et al., 2015). There is a large literature of prototype vaccines engineered by plant biotechnology for inclusion in food or feed especially for situations requiring mass immunization at minimal cost (Daniell et al., 2009). Plant biopharma is well matched to the needs and economic limitations of aquaculture (Clarke et al., 2013). Soybean feed-based vaccines can meet this need as a stacked trait. Soybean can be induced to produce up to 1% of its total protein shown as a prototype vaccine (Piller et al., 2005; Moravec et al., 2006). To produce greater levels of vaccine proteins technology has been developed to produce high-levels of heterologous proteins in soybean (Schmidt and Herman, 2008). An engineering strategy has been developed where transgenes encoding heterologous proteins can be produced at high levels, >8% of the total protein, by mimicking the gene of a compensating glycinin. Using this type of technology it should be feasible to design feed vaccines that separately or simultaneously produce several antigens potentially stacked with a strong adjuvant such as the enterotoxin LTB (Moravec et al., 2006) that could be produced in different formulations to vary and stagger the sensitization. This approach may improve the performance of oral vaccines for aquaculture.

### Carotenoid Enhancement

Astaxanthin (3,3′-dihydroxy 4,4′-diketo-β-carotene) is the carotenoid responsible for giving many crustaceans and some bird species their signature pink/red color. Its principle commercial use is a flesh colorant for fish. Farm-raised salmonoid fish are deprived of phytoplankton and/or algae that would naturally provide this colorful compound, making it necessary to supplement fish diet with additive pigments in order to ensure that the resultant filets appeal to consumers. In the U.S. alone, astaxanthin sales per year are about $200 million for just the salmon aquaculture industry (Lorenz and Cysewski, 2000). In order for plants to be an effective sustainable supply of this colorant it would have be both biosynthesized and accumulated at relevant levels and produced in a suitable delivery plant organ system. Proof-of-concept in the successful production of astaxanthin has been achieved in tobacco nectar (Mann et al., 2000; Ralley et al., 2004) and leaves (Hasunuma et al., 2008) and tubers of both potato (Gerjets and Sandmann, 2006) and carrot (Jayaraj et al., 2008). The highest level obtained was 64 μg astaxantin/g of the nectar of transgenic tobacco plants. Production of this carotenoid in seeds has been shown to be possible but with marginal accumulation levels: transgenic canola (*Brassica napus*) seeds contained 0.2 μg astaxanthin/g (Fujisawa et al., 2009) and transgenic soybean seeds 7 μg astaxanthin/g dry seed (Pierce et al., 2015).

**FIGURE 2:**
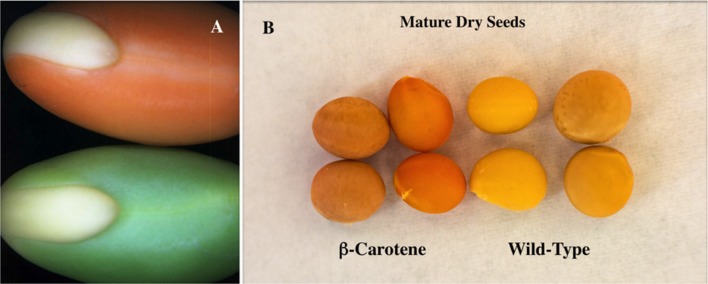
**β-carotene biofortified transgenic soybean seeds. (A)** developing soybean cotyledons from (bottom) non-transgenic soybean compared to (top) chloroplast localized seed specific bacterial phytoene synthase transgenic soybean. (**B**; column 4) dry non-transgenic soybean seeds, (column 3) dry non-transgenic soybean seeds without seed coat, (column 2) dry transgenic soybean seeds without seed coat and (column 1) dry transgenic β-carotene soybean seeds. Enhanced β-carotene accumulation in transgenic soybean seeds provides a visually obvious orange color in both developing cotyledons and dry seeds.

Variations in the amount of astaxanthin produced in different plant systems can be attributed to the inherent ability of certain plant organs to accumulate carotenoids, leaf and flower tissue more than seeds, and the catalytic attributes of the carotenoid biosynthetic enzymes. Enzymes in the carotenoid pathway have been reported to act upon an assortment of various substrates, often resulting in a mixture of carotenoids produced rather than a majority of a specific sought metabolite. Following on research showing that enhanced β-carotene levels could be achieved by the overexpression of a bacterial *Pantoea* phytoene synthase gene in soybean seeds (**Figure 2**) (Schmidt et al., 2015b), this construct was then combined individually with two ketolase genes from different sources: one from bacteria *Brevundimonas* spp and the other algae known for its high concentration of astaxanthin *Haematococcus pluvialis.* A mixture of carotenoids resulted with only one line using the bacterial ketolase producing detectable levels of astaxanthin (Pierce et al., 2015). Further emphasizing the importance of enzyme choice, three β-carotene ketolase enzymes were used from different sources in *Arabidopsis* leaves and found a wide variety of successful production of the desired astaxanthin carotenoid (Zhong et al., 2011). Up to 2 mg/g was produced when the ketolase gene from *Chlamydomonas*, compared to 0.24 mg/g when *Chlorella* was the gene source and non-detectable levels when *Haematococcus* ketolase gene was used. These findings emphasis the importance of enzyme selection in the biosynthesis of carotenoids giving promise to the future production of sustainable and commercially viable levels of this carotenoid pigment in a suitable plant delivery system.

### Feed-based Omega 3 Fatty Acids

Desired oil modifications in seeds for aquaculture use would include the omega 3 (ω3) fatty acids. These fatty acids have been shown to enhance fish growth rates (Tocher, 2015) and are one of the human health promoting constituents in fish (for review, Calder and Yaqoob, 2009). Many plant seed oils are abundant in the ω-3 fatty acid α linolenic acid (18:3^Δ9,12,15^) yet the health promoting long chain ω3 fatty acids are more limiting in the human diet with their principle source being marine fish. Although a vital component to both human and marine life nutrition, long chain ω-3 fatty acids eicosapentaenoic (EPA 20: 5^Δ5,8,11,14,17^) and docosahexaenoic acid (DHA 22:6^Δ4,7,10,13,16,19^) must be consumed in diets due to human and fish being very limited in their ability to produce them (Williams and Burdge, 2006). The most successful research to produce DHA and EPA in oil seeds crops was reported in *Brassica* seeds and used nine introduced gene expression cassettes using fatty acid modifying enzymes from various marine sources to achieve 0.2% DHA and up to 15% EPA of total fatty acids (Wu et al., 2005). Research to produce these lipid compounds in plants has moved toward soon being able to make commercially viable levels for the aquaculture community.

## Soybeans in Functional Feed Will Meet an Urgent Need to Support the Growing Aquaculture Industry

*Triple Null* (*p34, kti, le*) is a potential platform in a path to stack additional transgene traits for functional aquafeed. The growing aquaculture industry and global demands for high quality animal protein will require the optimization of feed performance. Over the past two decades biotechnology has had a remarkable role in increasing yield and productivity. The next frontier is to enhance the performance of crops for specific end uses. The rapidly growing aquaculture industry over-dependent on unsustainable fishmeal sources is an ideal circumstance to explore the potential to design high performance functional feed ingredients.

## Author Contributions

This data contained and production of this manuscript is a collaborative effort of both MS and EH who contributed to all aspects of the manuscript’s production.

## Conflict of Interest Statement

The authors declare that the research was conducted in the absence of any commercial or financial relationships that could be construed as a potential conflict of interest. The reviewer AC and handling Editor declared their shared affiliation, and the handling Editor states that the process nevertheless met the standards of a fair and objective review.
